# Spatial and temporal tracking of multi-layered cells sheet using reporter gene imaging with human sodium iodide symporter: a preclinical study using a rat model of myocardial infarction

**DOI:** 10.1007/s00259-024-06889-2

**Published:** 2024-08-29

**Authors:** Kentaro Otani, Tsutomu Zeniya, Hidekazu Kawashima, Tetsuaki Moriguchi, Atsushi Nakano, Chunlei Han, Shunsuke Murata, Kunihiro Nishimura, Kazuhiro Koshino, Kenichi Yamahara, Masayuki Inubushi, Hidehiro Iida

**Affiliations:** 1 Department of Molecular Pharmacology, National Cerebral and Cardiovascular Center Research Institute, Osaka, Japan; 2 Graduate School of Science and Technology, Hirosaki University, Aomori, Japan; 3 Radioisotope Research Center, Kyoto Pharmaceutical University, Kyoto, Japan; 4 Tandem Accelerator Complex (UTTAC), University of Tsukuba, Ibaraki, Japan; 5 Department of Research Promotion and Management, National Cerebral and Cardiovascular Center Research Institute, Osaka, Japan; 6 Turku PET Centre, Turku University Hospital, Turku, Finland; 7 Department of Preventive Medicine and Epidemiology, National Cerebral and Cardiovascular Center Research Institute, Osaka, Japan; 8 Department of Systems and Informatics, Hokkaido Information University, Hokkaido, Japan; 9 Laboratory of Molecular and Cellular Therapy, Institute for Advanced Medical Sciences, Hyogo Medical University, Hyogo, Japan; 10 Division of Nuclear Medicine, Department of Radiology, Kawasaki Medical School, Okayama, Japan; 11 Turku PET Centre, University of Turku and Turku University Hospital, Building 14, Kiinamyllynkatu 4-8, Turku, 20520 Finland

**Keywords:** Cells sheet, Mouse embryonic fibroblast, Myocardial infarction, Reporter imaging, SPECT

## Abstract

**Purpose:**

This study aimed to evaluate a novel technique for cell tracking by visualising the activity of the human sodium/iodide symporter (hNIS) after transplantation of hNIS-expressing multilayered cell sheets in a rat model of chronic myocardial infarction.

**Methods:**

Triple-layered cell sheets were generated from mouse embryonic fibroblasts (MEFs) derived from mice overexpressing hNIS (hNIS-Tg). Myocardial infarction was induced by permanent ligation of the left anterior descending coronary artery in F344 athymic rats, and a triple-layered MEFs sheets were transplanted to the infarcted area two weeks after surgery. To validate the temporal tracking and kinetic analysis of the transplanted MEFs sheets, sequential cardiac single-photon emission computed tomography (SPECT) examinations with a ^99m^TcO_4_^–^ injection were performed. The cell sheets generated using MEFs of wild-type mice (WT) served as controls.

**Results:**

A significantly higher amount of ^99m^TcO_4_^–^ was taken into the hNIS-Tg MEFs than into WT MEFs (146.1 ± 30.9-fold). The obvious accumulation of ^99m^TcO_4_^–^ was observed in agreement with the region where hNIS-Tg MEFs were transplanted, and these radioactivities peaked 40–60 min after ^99m^TcO_4_^–^ administration. The volume of distribution of the hNIS-Tg MEF sheets declined gradually after transplantation, implying cellular malfunction and a loss in the number of transplanted cells.

**Conclusion:**

The reporter gene imaging with hNIS enables the serial tracking and quantitative kinetic analysis of cell sheets transplanted to infarcted hearts.

**Supplementary Information:**

The online version contains supplementary material available at 10.1007/s00259-024-06889-2.

## Introduction

Chronic heart failure caused by myocardial infarction (MI) is a life-threatening disease worldwide [1]. To recover cardiac function using a regenerative treatment approach, several stem cell-based therapies have been developed [2]. Stem cells transplanted as cell suspensions into an infarcted heart exhibit low cell retention and poor cell survival. To overcome this issue, cell sheet technologies have been developed and shown to improve the efficiency of stem cell-based therapies for ischaemic heart disease [3].

Understanding the cell dynamics after transplantation is important for stem cell therapy. In the past two decades, several strategies, including magnetic resonance imaging (MRI), optical imaging, nuclear imaging, and histological analysis, have been applied to track transplanted stem cells in cardiovascular research [4, 5]. Although cell tracking using MRI has a high spatial and temporal distribution for detecting transplanted cells, the persistence of iron nanoparticles, even after the death of the transplanted cells, disturbs the temporal tracking of transplanted cells [6, 7]. Optical imaging also has several limitations, including poor tissue penetration of signals, low spatial resolution, and difficulties in 3-dimentional recognition of transplanted cells [4]. Additionally, histological analysis should not be relied upon for the repetitive assessment of transplanted cells in the heart of an individual, as it necessitates euthanizing the recipient animal. Therefore, an alternative approach that enables the repetitive and noninvasive assessment of viable cell dynamics is highly desirable for tracking surviving stem cells in cardiovascular research.

Sodium/iodide symporter (NIS; aka SLC5A5), an intrinsic transmembrane glycoprotein, mediates the transport of sodium and iodide ions in the mammalian thyroid, salivary glands, stomach, and small intestine [8, 9]. Recently, human NIS (hNIS) has been used as a human-derived reporter gene to track intramyocardially injected cells, and clearly visualized NIS-expressing cells have been demonstrated using the nuclear medicine technique [10, 11]. Both modalities of single-photon emission computed tomography (SPECT) and positron emission tomography (PET) could be used by combining them with radiotracers of ^123^I, ^125^I, ^131^I, and ^99m^TcO_4_^–^ for SPECT and ^124^I, and ^18^F-labeled Tetrafluoroborate (^18^F-TFB) for PET [12]. Although SPECT has poorer spatial resolution with lower sensitivity than PET, image quality appeared to be reasonably good, particularly when using ^99m^TcO_4_^–^ largely attributed to its high accumulation through the hNIS, which is beneficial due to its versatility and lower cost for radioactive nuclides. Moreover, other attractive features of ^99m^TcO_4_^–^ are attributed to the emitting photon energy of 140 keV as a single peak with a half-life of 6.0058 h. The energy could be close to ideal because the photons are not attenuated too large in the body, and the detection efficiency is reasonably high using a standard NaI scintillation detector.

Moreover, recent works demonstrated that volumetric images can be accurately reconstructed when providing a complete set of projection data in Radon Transform [13]. Quantitative parametric images such as the myocardial perfusion in units of ml/min/ml could also be calculated using existing clinical SPECT systems following adequate mathematical models and appropriate corrections for attenuation and scatter [14–16]. Recently, novel technologies have become available in clinical environments involving the CZT-detector systems focussing on the myocardial region [17], the use of CZT-detector for the cylindrical arrangement enabling increased sensitivity with an improved spatial resolution [18], and the use of double focus fan-beam collimator to increase the sensitivity in the heart region [19, 20]. These might further enhance the reality in visualization and quantitative assessment of hNIS-expressing transplanted myocardial cells in the clinical setting.

Under these circumstances, we aimed to (1) examine the feasibility of tracking the surviving cells transplanted to the myocardium with an old MI using SPECT with hNIS in a rat model and (2) examine the potential of the kinetic analysis approach in terms of quantifying the transport and membrane potential functions in the transplanted cell sheet using SPECT with hNIS.

## Materials and methods

### Animals

All animal studies were approved by the Institutional Animal Care and Use Committee of the National Cerebral and Cardiovascular Center and conducted in accordance with the guidelines of the Physiological Society of Japan. Mice and rats were group-housed under a 12/12-hour light/dark cycle at 25°C and had unrestricted access to food and water. Mice overexpressing hNIS (hNIS-Tg mice) were generated as previously described [21]. Briefly, hNIS cDNA was cloned into a pCAGGS vector, and the linearised transgene construct was transferred into fertilised eggs of C57BL/6 N mice by microinjection [22]. For genotyping, genomic DNA was extracted from mouse tails using a DNeasy Blood & Tissue Kit (#69504; Qiagen K.K., Tokyo, Japan). Two sets of polymerase chain reaction (PCR) primers for detecting the 5’- and 3’-ends of hNIS transgene were as follows: forward: 5’-ATGGAGGCCGTGGAGAC-3’, reverse: 5’-CGGTGAAGAAGTCCTCAGC-3’ for 5’-end; forward: 5’-CCTGCCCACCAATGAGG-3’, reverse: 5’-TCAGAGGTTTGTCTCCTGCTG-3’ for 3’-end.

Female C57BL/6 N (wild-type; WT) mice bred at Japan SLC Inc. (Shizuoka, Japan) were used to obtain the hNIS-Tg and WT embryos. For the rat MI model, 8-week-old male F344 athymic nude (rnu/rnu) rats were purchased from CLEA Japan, Inc. (Tokyo, Japan). The allocation of the animals used is summarised in Table 1.

**Table 1:** Summary of animal information

Experiments	Animal		BW (g)	Cells sheet	Imaging date	Dose (MBq)	SPECT protocol
	No.	Age (wk)					
Whole-body imaging	WT	6	20.0	–	–	185.0	Figure 1A
	Tg	6	17.4	–	–	218.3	Figure 1A
Validation of cells sheet detection	Rat 1	11	230	hNIS-Tg	Day 4	412.2	Figure 1B
	Rat 2	11	220	hNIS-Tg	Day 4	529.6	Figure 1B
	Rat 3	11	227	hNIS-Tg	Day 4	496.7	Figure 1B
	Rat 4	16	245	hNIS-Tg	Day 4	505.5	Figure 1B
	Rat 5	17	263	hNIS-Tg	Day 4	521.5	Figure 1B
	Rat 6	13	241	hNIS-Tg	Day 4	520.9	Figure 1B
	Rat 7	14	221	hNIS-Tg	Day 4	524.7	Figure 1B
	Rat 8	12	223	WT	Day 4	509.5	Figure 1B
	Rat 9	14	246	WT	Day 4	472.7	Figure 1B
Histological analysis	Rat 10	11	239	hNIS-Tg	Day 4	–	–
Serial tracking of cell sheet in the same individual	Rat 11	11	182	hNIS-Tg	Day 1	662.0	Figure 1C
		11	180		Day 4	454.4	Figure 1C
		12	182		Day 7	508.0	Figure 1C
		12	185		Day 10	469.0	Figure 1C
		13	195		Day 15	384.2	Figure 1C
		14	216		Day 23	476.5	Figure 1C

WT; wild-type mouse, Tg; hNIS-transgenic mouse, BW; body weight

### Isolation and culture of mouse embryonic fibroblasts (MEFs)

Female WT mice were crossed with male hNIS-Tg mice, and the day after cohabitation was defined as E0.5. Primary hNIS-Tg or WT MEFs were isolated from E13.5 embryos as previously reported [23] with minor modifications. The detailed description can be found in the Supplementary Method. Throughout the study, MEFs at passage 3–5 were used.

### Semi-quantitative reverse transcription PCR

hNIS mRNA expression in MEFs was quantified by semi-quantitative reverse transcription PCR. The detailed description can be found in the Supplementary Method.

### Immunocytochemistry of hNIS

The subcellular distribution of the hNIS protein in hNIS-Tg MEFs was examined by immunocytochemistry, as reported previously [24], with minor modifications. The detailed description can be found in the Supplementary Method. Fluorescent images were acquired using the BIOREVO BZ-9000 microscope (Keyence Corporation, Osaka, Japan).

### ^99m^TcO_4_^–^uptake assay

To examine the function of the hNIS protein in hNIS-Tg MEFs, ^99m^TcO_4_^–^ uptake assay was performed as previously described [25]. The detailed description can be found in the Supplementary Method. The percentage of ^99m^TcO_4_^–^ uptake was calculated by dividing the radioactivity of cell lysates by the total amount of radioactivity. The uptake of ^99m^TcO_4_^–^ by MEFs was also examined under conditions of co-treatment with 50 µM sodium perchlorate (NaClO_4,_ #198–09252, FUJIFILM Wako Pure Chemical), a competitive inhibitor of hNIS. Assays were performed in quadruplicate using five WT and hNIS-Tg MEFs lines.

### Myocardial infarction model

F344 rnu/rnu rats (*n* = 18) were anaesthetised by intraperitoneal injection of pentobarbital sodium (50 mg/kg), intubated, and mechanically ventilated with a rodent ventilator in room air. Through a left lateral thoracotomy, the left anterior descending coronary artery was ligated with a 6 − 0 polypropylene suture (NESCOSUTURE^®^, Alfresa Pharma Corporation, Osaka, Japan). To exclude an insufficient MI model, the cardiac function of postoperative rats was assessed qualitatively by echocardiography one week after surgery. Five rats died within 2 weeks of the surgical procedure, and another two rats were excluded from the experiment because of an insufficient MI model. Therefore, 11 rats were enrolled in the study.

### MEFs sheets transplantation

Two weeks after the surgical procedure, triple-layered MEF sheets were transplanted into the infarcted rat heart. The preparation and stacking of MEF sheets were performed as described previously [26]. The detailed description can be found in the Supplementary Method. Consequently, 9 hNIS-Tg and 2 WT MEFs sheets were prepared.

### SPECT/CT imaging

A commercially available 4-head multiplexing multi-pinhole SPECT system (BioScan NanoSPECT/CT; Mediso Medical Imaging Systems, Budapest, Hungary) was used [27]. Two types of multi-pinhole collimator sets were applied: APT3 (pinhole diameter; 1.0 mm, number of pinholes/head, 9) for mice and APT5 (pinhole diameter; 1.5 mm, number of pinholes/head, 9) for rats with MI. SPECT images were acquired using Nucline™ software (Mediso) and reconstructed using HiSPECT software (SciVis GmbH, Göttingen, Germany). ^99m^TcO_4_^–^ eluted from a ^99^Mo–^99m^Tc generator (Meditec^®^; Nihon Medi-Physics Co., Ltd, Tokyo, Japan) was used as the radiotracer. An additional examination was carried out to investigate the presence of the eluted ^99m^TcO_4_^–^ formed as colloids in the injection batch. The radio-TLC technique (matrix: silica gel, eluent: MeOH/water = 85/15) was used.

Prior to the animal studies, the selection of the multi-pinhole collimator, rotation radius that determines the transaxial field of view (FOV), and angular sampling steps were optimised to minimise overlap in the projection data. The accuracy of the reconstructed images was evaluated using a rat-sized uniform cylinder and mini-Derenzo phantoms filled with ^99m^Tc solution.

#### Experiment 1

**Fig. 1:**
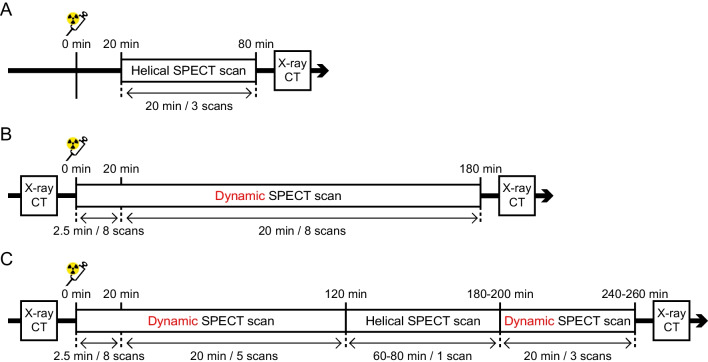
Experimental protocols** A** The protocol for whole-body SPECT/CT imaging in wild-type (WT) and hNIS-transgenic (hNIS-Tg) mice. **B** The SPECT/CT imaging protocol for examining the feasibility of the detection of the hNIS-Tg mouse embryonic fibroblasts (MEFs) sheets transplanted onto the infarcted rat heart (Day 4). **C** The SPECT/CT imaging protocol for examining the temporal tracking of transplanted hNIS-Tg MEFs sheets on the infarcted rat heart (Day 1–Day 23)

The whole-body organ distribution of ^99m^TcO_4_^–^ was examined in 6-week-old WT and hNIS-Tg mice. Mice were anaesthetised with isoflurane and placed in the prone position. After a scout view (45 kVp, 500 msec), the helical SPECT scan was started 20 min after intravenous administration of ^99m^TcO_4_^–^ through the tail vein (185.0 MBq for WT, 218.3 MBq for hNIS-Tg mouse) using the following settings: scan FOV, 30 mm in diameter and 86 mm in length; number of projections, 24; time per projection, 40 s; duration of the scan, 20 min; and number of scans, 3. CT images were obtained after the completion of SPECT imaging (45 kVp, 0.177 mA). After completion of the SPECT/CT experiments, whole blood was collected from each mouse, and radioactivity was measured using a well counter (BeWell-QS02, Molecular Imaging Laboratories, Inc., Osaka, Japan). The protocol for whole-body SPECT/CT is shown in Fig. 1A.

#### Experiment 2

SPECT/CT imaging was performed in nine rats with MI 4 days after cell sheet transplantation to examine the feasibility of detection and kinetic analysis of the transplanted hNIS-Tg MEFs sheets (Rats #1–#9 in Table 1). After a scout view and CT images (65 kVp, 0.123 mA) were completed, a sequential dynamic SPECT scan was initiated (2.5 min×8 scans at 20 s/projection and 20 min×8 scans at 195 s/projection) at the same time with the ^99m^TcO_4_^–^ administration (412.2–529.6 MBq / animal) via a tail vein. The scan FOV was 60 × 24 mm and the angular sampling step was set at 15 degrees (24 projections). The left femoral artery was catheterised using a PE50 polyethylene tube to monitor the blood pressure and heart rate. The experimental protocol is shown in Fig. 1B.

#### Experiment 3

The feasibility of the temporal tracking of hNIS-Tg MEFs sheets using SPECT was examined (Rat #11 in Table 1). The experimental protocol is shown in Fig. 1C. In *Experiment 3*, we incorporated the helical SPECT scan in the experimental protocol to examine the ^99m^Tc accumulations in multi-organ. However, only the heart region was analyzed in the present study. Thus, the data quality is essentially identical between Experiments 2 and 3. SPECT settings were almost identical to those used in Experiment 2. The settings of the helical SPECT scan were as follows: scan FOV, 60 mm in diameter and 90–114 mm in length, with 24 projections at 195 s/projection. SPECT/CT experiments were performed on days 1, 4, 7, 10, 15, and 23 after the hNIS-Tg MEFs sheets transplantation. Administered ^99m^TcO_4_^–^ was 384.2–662.0 MBq / animal.

### Ratio of radioactivity between plasma and whole blood

The ratio of radioactivity between plasma and whole blood was serially examined using three additional male F344 rnu/rnu rats (253 ± 5 g), as reported previously [16]. In brief, arterial blood samples were collected at 2.5, 5, 10, 20, 40, 60, 120, and 180 min after ^99m^TcO_4_^–^ injection (483.5–511.3 MBq / animal). Plasma was separated immediately after sampling by centrifugation, and the radioactivity of the plasma and whole blood samples was measured in a well counter cross-calibrated using a SPECT scanner (BeWell-QS02, Molecular Imaging Laboratories). The ratio of radioactivity between plasma and whole blood was calculated by averaging throughout the experimental period.

### Consecutive monitoring of radioactivity in the arteriovenous shunt

To validate the arterial input function (AIF) determined from SPECT images, AIF was also acquired using an arteriovenous shunt (AVS), as reported previously [28]. In brief, the right femoral artery and vein were catheterised using PE50 tubes 55 and 35 cm in length, respectively. To form the AVS, 10 cm from the ends of the two tubes were connected to a PE100 tube. The radioactivity on the arterial side of the PE50 tube was measured using a Gd_2_SiO_5_:Ce (GSO) detector (GSO input function monitor system; Molecular Imaging Laboratory). The patency of the AV shunt was confirmed in part of the PE100 tube using a portable ultrasound system (LOGIC BooK XP, GE Healthcare Japan, Tokyo, Japan) with an i12L-RS transducer.

### Data analysis

In the ^99m^TcO_4_^–^ uptake study, the intracellular uptake of ^99m^TcO_4_^–^ after 5, 30, and 60 min of incubation was compared between the WT and hNIS-Tg MEFs. Additionally, the inhibitory effect of NaClO_4_ on the intracellular uptake of ^99m^TcO_4_^–^ was examined in WT and hNIS-Tg MEFs.

SPECT images were analysed using the VivoQuant 3.5 software (Invicro. LLC., Needham, MA, USA) with a voxel size of 0.5 × 0.5 × 0.5 mm^3^. In the case of a fused image with the CT image, the voxel size was 0.4 × 0.4 × 0.4 mm^3^. Representative SPECT images of each animal are presented as the standard uptake value (SUV) by dividing the radioactivity by the injected dose and body weight.

Quantitative analysis and model fitting of the time-activity curve were performed using Carimas software (Turku PET Centre, Finland). The volumes of interest (VOI) were set on the left ventricular cavity (LV), hNIS-Tg MEF sheets, and normal myocardium, with average sizes of 406 voxels (356–439), 104 voxels (47–146), and 96 voxels (82–119), respectively. The decay-corrected time-activity curve data determined by SPECT and AVS were multiplied by the ratio of radioactivity between the plasma and whole blood. Kinetic modelling was performed using a one-tissue compartment model to estimate the quantitative kinetic parameters: ***K***_1_ (a rate constant for transfer from arterial plasma to tissue), ***k***_2_ (a rate constant for transfer from tissue to arterial plasma), and ***V***a (the arterial blood volume). The total volume of distribution (***V***_T_) was calculated by dividing ***K***_1_ by ***k***_2_. In *Experiment 3*, both first and second dynamic SPECT data were incorporated for kinetic model fitting. Additionally, we adopted the bootstrap approach [29, 30] to quantify the uncertainty in the model fitting of the time-activity curve in the area of the transplanted hNIS-Tg MEFs sheet.

### Histological analysis

An additional nine-week-old male F344 rnu/rnu rats subjected to MI were treated with triple-layered hNIS-Tg MEFs sheets and euthanised 4 days after transplantation by an overdose of an anaesthetic agent, and the heart was removed (Rat #10). The excised heart was fixed with 4% paraformaldehyde (FUJIFILM Wako Pure Chemical Corporation) at 4 °C overnight, embedded in paraffin, and cut into 2-µm sections. The sections were stained with haematoxylin and eosin to delineate the transplanted MEFs sheets.

### Statistical analysis

The data are presented as the mean ± the standard error of the mean unless otherwise stated. Pairwise comparisons were performed using two-tailed unpaired Student’s *t*-test. Differences among three or more groups were analysed using a two-way analysis of variance (ANOVA) with the Tukey–Kramer post-hoc test. A *statistical* significance was defined as *P* < 0.05. The detailed results of the two-way ANOVA are summarised in Supplementary Table.

## Results

### Characterisation of hNIS-Tg MEFs

Three hNIS-transgenic mouse lines were established. hNIS-Tg mouse lines that stably expressed the transgene were obtained by maintaining successive brother-sister mating for more than 20 generations. As there were no significant differences in the ^99m^TcO_4_^–^ uptake among the three hNIS-Tg mouse lines (data not shown), one line was selected and used for this study.

**Fig. 2:**
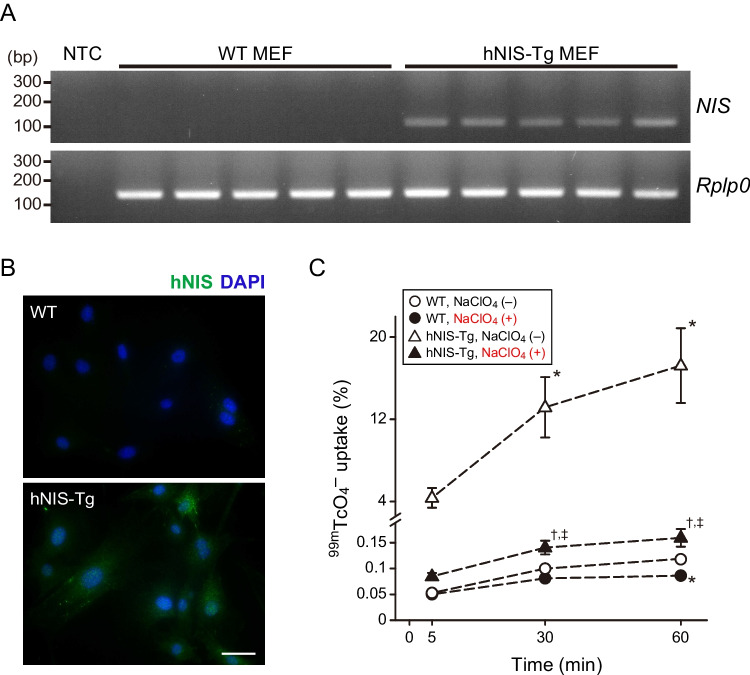
Characteristics of the hNIS-transgenic (hNIS-Tg) mouse embryonic fibroblasts (MEFs) **A** The expression of the hNIS in the wild-type (WT) and hNIS-Tg MEFs. NTC, No-template control. **B** Immunocytochemistry for NIS in the WT and hNIS-Tg MEFs. Scale bar, 50 μm. **C** Summary of the in vitro ^99m^TcO_4_^–^ uptake assay using WT and hNIS-Tg MEFs. Statistical analysis was performed using a two-way analysis of variance with the Tukey–Kramer post hoc test. *P < 0.05 versus WT, NaClO_4_ (–), ^†^*P* < 0.05 versus hNIS-Tg, NaClO_4_ (–), ^‡^*P* < 0.05 versus WT, NaClO_4_ (+). *n* = 5 per group

Figure 2A shows the hNIS mRNA expression, as determined by semi-quantitative PCR. Uncropped gel images are shown in Supplementary Fig. S1. hNIS-Tg MEFs expressed abundant hNIS mRNA, while it was difficult to observe in WT MEFs.

The subcellular distribution of hNIS as determined by immunocytochemistry is shown in Fig. 2B. The hNIS protein was expressed ubiquitously in the cytoplasm of hNIS-Tg MEFs, whereas no obvious hNIS expression was detected in the WT MEFs.

Figure 2C shows the time-dependent changes in ^99m^TcO_4_^–^ uptake in WT and hNIS-Tg MEFs. In WT MEFs, the intracellular uptake of ^99m^TcO_4_^–^ was increased slightly in a time-dependent manner. The intracellular uptake of ^99m^TcO_4_^–^ was markedly greater in the hNIS-Tg MEFs than that in the WT MEFs. The average rate of increase of ^99m^TcO_4_^–^ uptake in hNIS-Tg MEFs over WT MEFs after 60 min of incubation was 146.1 ± 30.9-fold. The NaClO_4_ treatment significantly decreased the intracellular uptake of ^99m^TcO_4_^–^ both in the WT and hNIS-Tg MEFs. However, the intracellular uptake of ^99m^TcO_4_^–^ under conditions of co-treatment with NaClO_4_ was still higher in the hNIS-Tg MEFs than in WT MEFs.

### Whole-body SPECT images in mice

**Fig. 3:**
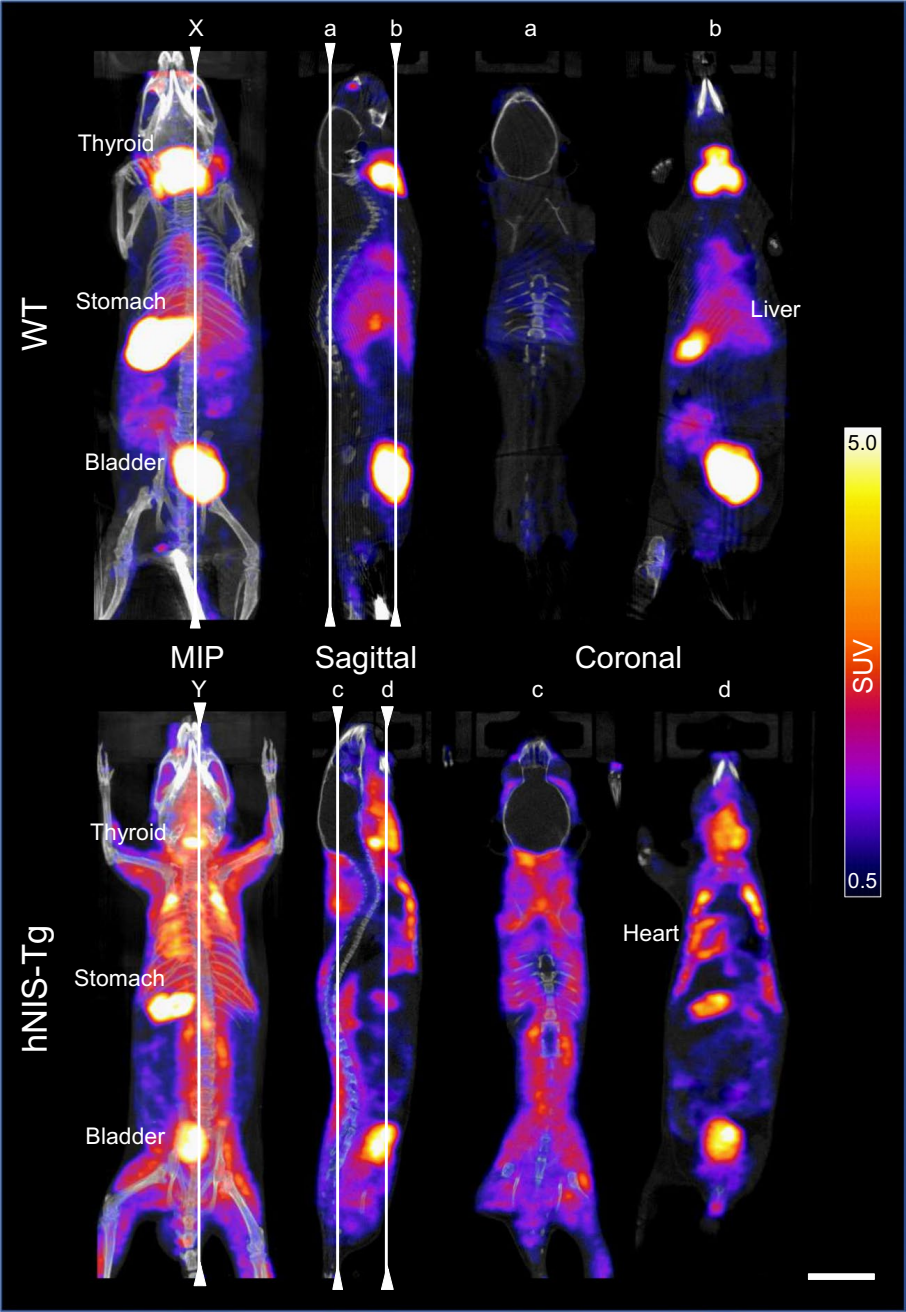
Comparison of the intracellular uptake of^99m^TcO_4_^– ^between the wild-type (WT) and the hNIS-transgenic (hNIS-Tg) mouse Two vertical lines (X and Y) in the MIP images indicate the locations of the sagittal images. Four vertical lines (a through d) in the sagittal view indicate the locations of the coronal images. Scale bar, 10 mm. MIP; maximum intensity projection, SUV; standardised uptake value

The radio-TLC analysis did not demonstrate the presence of the hydrolyzed-reduced ^99m^Tc-labelled colloids as shown in Supplementary Fig. S2. Figure 3 and Supplementary Movies S1 and S2 show the whole-body distribution of ^99m^TcO_4_^– ^in the WT and hNIS-Tg mice. The radioactivity accumulation was particularly clear in the thyroid, stomach, and bladder of the WT mouse (upper panel of Fig. 3), whereas a homogenous distribution of ^99m^TcO_4_^–^ in the skeletal muscles and myocardium was found in the hNIS-Tg mouse (lower panel of Fig. 3). After completion of SPECT/CT imaging, the radioactivity in the whole blood was measured. The percent injected dose per gram (%ID/g) of hNIS-Tg whole blood was lower than that of WT whole blood (0.30% vs. 3.95%).

### SPECT/CT imaging of the transplanted hNIS-Tg MEFs sheets on the infarcted rat heart

By means of the selections of the multi-pinhole collimator (APT-5), the transaxial FOV of 60 mmΦ, and the angular step of 15º, the overlap of projection data reached a negligible level for the 30 mm diameter cylindrical phantom. The Gaussian postfilter has a maximum resolution of 1.3 mm. The reconstructed images also demonstrated a uniform distribution for the uniform cylindrical phantom and a good separation of multiple line sources for the mini-Derenzo phantom. The SPECT images of rats reconstructed with the same settings as those used for the phantom experiments resulted in reasonable image quality in all animal experiments.

**Fig. 4:**
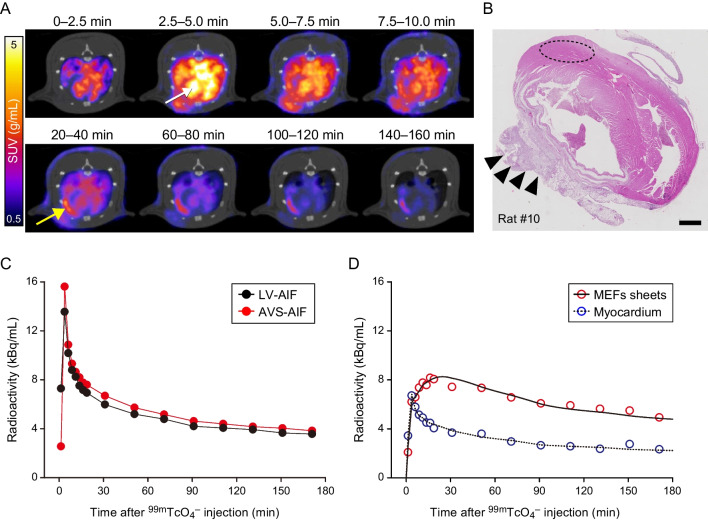
Sequential examination of the infarcted rat heart treated with hNIS-transgenic mouse embryonic fibroblasts (hNIS-Tg MEFs) sheets by SPECT/CT imaging with^99m^TcO_4_^– ^injection **A **Serial changes in the SPECT/CT images of infarcted rat heart at 4 days after hNIS-MEFs sheets transplantation. The white and yellow arrows in the figure indicate the left ventricular cavity (LV) and hNIS-Tg MEFs sheets, respectively. SUV; standardised uptake value. **B** Haematoxylin and Eosin staining of rat heart treated with the hNIS-Tg MEFs sheets (Rat #10). The arrow heads and dotted ellipse in the figure indicate the transplanted MEFs sheets and volume of interest on the myocardium, respectively. Scale bar, 1 mm. **C** Comparison of arterial input function (AIF) derived from arteriovenous shunt (AVS) and that determined from volume of interest in LV. The rat is the same as in **A**. **D** A representative time-radioactivity curve of the hNIS-Tg MEFs sheets and myocardium. The solid and dotted lines in the figure depict the fitting curve. The rat is the same as in **A**

Representative serial transverse SPECT/CT images of infarcted rat hearts four days after hNIS-Tg MEFs sheets transplantation are shown in Fig. 4A (Rat #5). An obvious accumulation of ^99m^Tc was observed on the surface of the rat heart 20 min after injection. Figure 4B shows the haematoxylin and eosin staining of infarcted rat hearts treated with hNIS-Tg MEFs sheets (Rat #10). The spatial distribution of the MEFs sheets transplanted to the infarcted heart aligned well with the accumulation of ^99m^TcO_4_^–^ as determined by SPECT imaging. Taken together, the accumulation of ^99m^TcO_4_^–^ observed on the surface of infarcted rat hearts appears to originate from the transplanted hNIS-Tg MEFs sheets.

The ratio of radioactivity between the plasma and whole blood is shown in Supplementary Fig. S3. Throughout the SPECT experiment, the ratio of radioactivity between plasma and whole blood was almost constant. The average plasma-to-whole blood ratio was 1.239 ± 0.048.

Figure 4C presents a comparison of the time-activity curves of the LV and AVS. The radioactivity in the LV reached a peak within several minutes after ^99m^TcO_4_^–^ injection but disappeared promptly thereafter (white arrow in Fig. 4A). The time-activity curve of the AVS was similar to that of the LV. While the radioactivity in the normal myocardium disappeared promptly after reaching the peak, the accumulation of ^99m^TcO_4_^–^ in the hNIS-Tg MEFs sheets was observed 20 min after ^99m^TcO_4_^–^ injection, and it remained for about 3 h after ^99m^TcO_4_^–^ administration (yellow arrow in Fig. 4A and D).

### Validation of the hNIS-Tg MEFs sheets detection on the infarcted rat heart

**Fig. 5:**
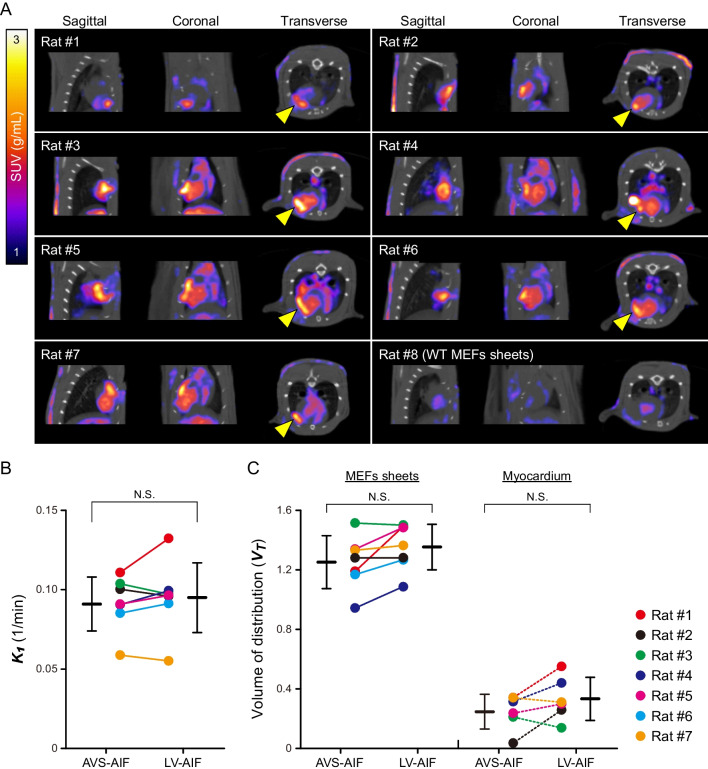
Reproducibility of the hNIS-Tg cells sheets detection using SPECT with^99m^TcO_4_^–^injection** A** Summary of the SPECT/CT images of the infarcted rat heart at 4 days after treatment with hNIS-transgenic (hNIS-Tg; Rat #1 through Rat #7) or wild-type (WT; Rat #8) mouse embryonic fibroblasts (MEFs) sheets. The yellow arrowheads in the figure indicate the hNIS-Tg MEFs sheets. **B** Comparison of the ***K***_***1***_ values calculated from the 1-tissue compartment model with two types of arterial input functions (AIFs): arteriovenous shunt (AVS) and left ventricle (LV). N.S. indicates not significant. **C** Comparison of the volume of distribution (***V***_T_) values calculated with two types of AIFs in the hNIS-Tg MEFs sheets and myocardium. The kinetic modelling was unable in myocardium of the Rat #6. The data are presented as the mean ± one standard deviation. N.S. indicates not significant

Figure 5A summarises the SPECT/CT images of infarcted rat hearts treated with hNIS-Tg MEFs or WT MEFs sheets 40–60 min after ^99m^TcO_4_^–^ injection. The accumulation of ^99m^TcO_4_^–^ was observed in all seven rats treated with the hNIS-Tg MEFs sheets, whereas it was not detected in the WT MEFs sheet-transplanted rats.

Figure 5B shows a comparison of ***K***_1_ values of the hNIS-Tg MEFs sheets determined using the radioactivity of LV or AVS as an AIF. Although there were some variations among the subjects, no obvious differences were found, even when each radioactivity was used as an input function (AVS-AIF: 0.091±0.006 vs. LV-AIF: 0.095±0.008). Figure 5C depicts the ***V***_T_ values of the hNIS-Tg MEFs sheets and myocardium determined by the two types of AIF. There were no significant differences, irrespective of the utilisation of each radioactivity as an AIF (AVS-AIF: 1.25±0.07 vs. LV-AIF: 1.35±0.06 in the hNIS-MEFs sheets, AVS-AIF: 0.25±0.04 vs. LV-AIF: 0.33±0.05 in the myocardium).

As shown in Supplementary Fig. S4A, an unexpected accumulation of ^99m^TcO_4_^–^ was observed in the thoracic cavity of rats implanted with either WT or hNIS-Tg MEFs sheets. At these unexpected accumulation sites, the ***K***_1_ value was much higher than those of the hNIS-Tg MEFs sheets (Fig. 5B). The SPECT image of the excised tissue showed that the ^99m^TcO_4_^–^ was accumulated not in the heart but in the lungs of WT MEFs sheet-transplanted rats (Supplementary Fig. S4B).

### Serial tracking of the hNIS-Tg MEFs sheets transplanted on the infarcted rat heart

**Fig. 6:**
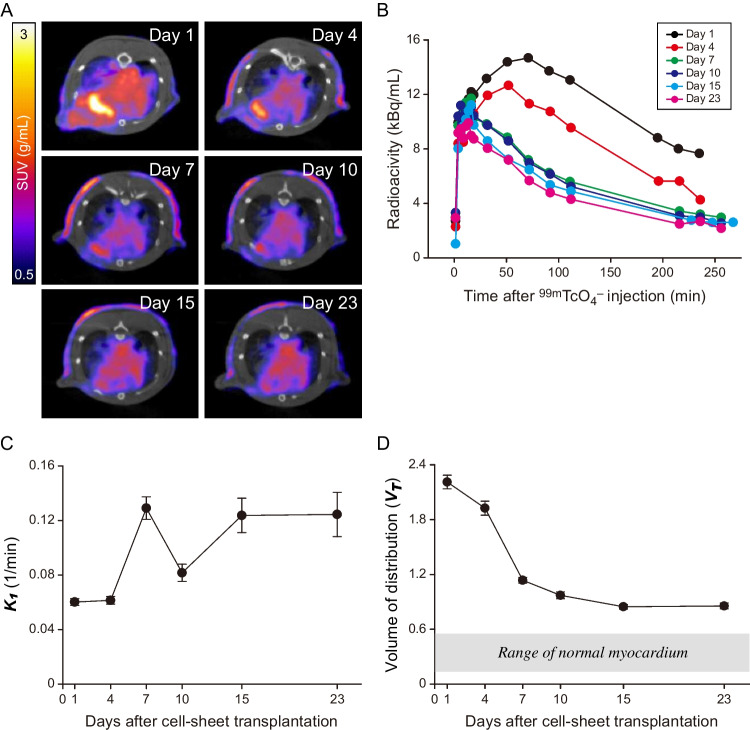
Temporal tracking of hNIS-Tg MEFs sheets transplanted to the infarcted rat heart using SPECT (**A**,** B)** Serial changes in the SPECT/CT images (**A**) and the time-activity curve of the hNIS-Tg MEFs sheets transplanted to the infarcted rat heart (**B**). The time-activity curves were normalised to the injected dose relative to Day 1. (**C**,** D**) Temporal changes in the ***K***_***1***_ value (**C**) and the volume of distribution (***V***_T_) value (**D**) of transplanted hNIS-Tg MEFs sheets. The error bards represent one standard deviation estimated by the bootstrap analysis. SUV; standardised uptake value

Temporal changes in SPECT/CT images of infarcted rat hearts treated with hNIS-Tg MEFs sheets are shown in Fig. 6A. The extent of ^99m^TcO_4_^–^ accumulation in the infarcted rat heart diminished daily after hNIS-Tg MEFs sheets transplantation. Figure 6B depicts the comparison of the time-activity curve of the hNIS-Tg MEFs sheets after transplantation. The time-activity curve of the hNIS-Tg MEFs sheets decreased in a time-dependent manner. Figure 6C shows the temporal changes in the ***K***_1_ value of the transplanted hNIS-Tg MEFs sheets. The ***K***_1_ value of the hNIS-Tg MEFs sheets tended to increase gradually until 15 days after transplantation; however, it became constant between days 15 and 23. Figure 6D depicts the temporal changes in the ***V***_T_ value of the hNIS-Tg MEFs sheets after transplantation. The ***V***_T_ decreased in a time-dependent manner and remained almost constant after 15 days. However, the ***V***_T_ value of the hNIS-Tg MEFs sheets 23 days after transplantation was still higher than that of the normal myocardium. This result implies that the transplanted hNIS-Tg MEFs remained intact even 23 days after transplantation. ***V***a did not indicate significant time dependency, and the average value over the whole experiment was 0.29 ± 0.06 ml/ml.

## Discussion

In the present study, we developed a transgenic mouse line that systemically expresses the human reporter gene, hNIS, under the control of the CAG promoter and demonstrated that hNIS-expressing cell sheets have the potential to reveal the cellular dynamics of transplanted cells in the infarcted heart. Additionally, we demonstrated the feasibility of using the time-activity curve of the LV as an alternative arterial input function. Transplanted hNIS-Tg MEFs were tracked visually for up to 15 days. Among the pharmacokinetic parameters, we demonstrated that ***V***_T_ is a robust parameter that reflects the number of remaining cells and their viability after cell transplantation, as ***V***_T_ declines in a time-dependent manner after transplantation.

The number of remaining living cells is a critical determinant for cell-based therapy; thus, temporal tracking of transplanted cells has been a challenge for the past decade [31, 32]. Two radiolabelling strategies (i.e., direct and indirect labelling) have been widely utilised for tracking transplanted cells by nuclear imaging [33]. Direct labelling of cells with radioisotopes before transplantation has high sensitivity; however, the half-life of radioisotopes hinders the long-term tracking of transplanted cells. Additionally, the acquired radioactivity data obtained from the directly labelled cells did not necessarily correspond to their viability. NIS-mediated indirect cell tracking has been demonstrated by several researchers in cardiovascular research [34, 35]. As the NIS-mediated intracellular transport of ^99m^TcO_4_^–^ is driven by the Na^+^ gradient generated by ATP hydrolysis by Na^+^/K^+^ ATPase [9], the serial functional parameters derived from ^99m^TcO_4_^–^-incorporated cells are thought to originate from living cells. Terrovitis et al. reported that NIS-positive cardiac-derived stem cells injected intramyocardially could be visualised by SPECT up to 6 days after cell injection in a rat MI model [10]. In contrast, Templin et al. reported that hNIS-positive human-induced pluripotent stem cells (iPSCs) can be tracked for up to 15 weeks in a swine-infarcted heart [11]. Because the retention and survival of transplanted cells in the infarcted heart are affected not only by the cell type [36] but also by the delivery route of the cell [37], it would be difficult to directly compare the duration of cell tracking. However, our results objectively demonstrated that the retention and survival of transplanted cells could be improved by combining cell sheet technology and that NIS-mediated reporter gene imaging could be a robust application for tracking the transplanted cells, irrespective of the cell type and delivery route.

In contrast to direct cell labelling, indirect labelling requires repeated injections of radioisotopes. In this study, SPECT imaging was adopted to track the transplanted hNIS-Tg MEF sheets sequentially; therefore, we needed to consider the half-life of radionuclides. Radioactive iodide also can be a candidate of radionuclides for visualizing hNIS-expressing cells using SPECT. However, considering the repeated SPECT imaging for a short period of time, the feature of a shorter half-life of ^99m^TcO_4_^–^ would be a big advantage for the choosing of radionuclides. Further studies using PET imaging might be desirable to examine the precise cellular dynamics after transplantation. Additionally, the targeted reporter genes should be stably expressed in cells for transplantation via indirect cell labelling. Primary mesenchymal stem cells (MSCs) isolated from various tissues have been widely used to treat cardiovascular diseases [38]. However, it is known that the functional properties of isolated primary MSCs depend on the donor’s characteristics, including age, health condition, and medication intake. [39]. Thus, the preparation of MSCs stably expressing NIS is an urgent matter for NIS-mediated cell tracking in cardiovascular research. Recent progress has enabled the generation of MSCs from human iPSCs [40, 41]. This technique has the potential to resolve both cellular quality for treatment and the stable expression of NIS for cell tracking.

The time-dependent decrease in the ***V***_T_ value of transplanted hNIS-Tg MEFs sheets reflects the lack of ^99m^TcO_4_^–^ retention inside the cell sheet due to cellular dysfunction or a decrease in cell number (Fig. 6D). To achieve precise and reproducible kinetic analysis of transplanted cells, it is desirable to distribute hNIS-Tg cells uniformly in the ischaemic area. The distribution of transplanted cells in the heart tends to be sparse after transendocardial, intramyocardial, or intravenous injections. Consequently, setting the VOI for kinetic analysis can be difficult. Conversely, the ^99m^TcO_4_^–^ accumulation was confined only to the heart surface when hNIS-Tg MEFs were transplanted as cell sheets (Fig. 4). Although the extent of ^99m^TcO_4_^–^ accumulation in the infarcted myocardium gradually decreased in a time-dependent manner, this locally confined feature facilitated the VOI setting (Fig. 6A). Furthermore, the uptake of ^99m^TcO_4_^–^ into the myocardium 40 min after injection was almost negligible (Fig. 4A and D**)**. Thus, the derived kinetic parameters would purely reflect the cellular kinetics of transplanted hNIS-Tg cells, even if the VOI contained some amount of myocardium. Although further validation studies in large animal models are required, reporter gene imaging targeting hNIS has the potential to lead to the development of clinically translatable kinetic analysis of transplanted cells.

This study had some limitations that need to be addressed. As shown in Fig. 5 and Supplementary Fig. S4, obvious ^99m^TcO_4_^−^ accumulation was observed in the lungs, even in a rat that received WT MEFs sheets transplantation. As we did not observe the hydrolyzed-reduced, colloidal form of ^99m^Tc in the eluted ^99m^TcO_4_^−^ (Supplementary Fig. S2), the ^99m^TcO_4_^−^ accumulation in the lungs could be due to tissue injury that might have been caused during the surgical procedure. Although it is unclear which cells in the lungs ^99m^TcO_4_^−^ was incorporated, the ***K***_1_ and ***V***_T_ values may be useful indices to judge the specificity of ^99m^TcO_4_^−^ uptake, as the ***K***_1_ and ***V***_T_ values in non-specific ^99m^TcO_4_^−^ uptake were much higher than those in the hNIS-Tg MEFs sheets. Further studies are needed to clarify the cell/tissue source of non-specific ^99m^TcO_4_^−^ uptake and eliminate its effect on kinetic analysis.

In conclusion, quantitative assessment of cell viability, cellular function, and dynamics of transplanted cells is feasible using hNIS-expressing cell sheets. SPECT is a commonly used modality in clinical settings, and gene-modified cell therapy has recently become popular in clinical settings (e.g., chimeric antigen receptor (CAR)-T therapy). Taken together, our results imply that hNIS-mediated reporter gene imaging could provide novel insights into stem cell therapy for cardiovascular diseases, not only in basic research, but also in clinical settings.

## Electronic supplementary material

Below is the link to the electronic supplementary material.

Supplementary movie S1 (GIF 2.32 MB)

Supplementary movie S2 (GIF 3.03 MB)

Supplementary file 1 (DOCX 10.5 MB)

## Data Availability

The datasets generated and/or analysed during the current study are available from the corresponding author upon reasonable request.
