# Supplementary figures and images for: Spatial and temporal tracking of multi-layered cells sheet using reporter gene imaging with human sodium iodide symporter: a preclinical study using a rat model of myocardial infarction
Source: Eur J Nucl Med Mol Imaging. 2024 Aug 29;52(1):74–87. doi: 10.1007/s00259-024-06889-2 (PMC11599416; doi:10.1007/s00259-024-06889-2)

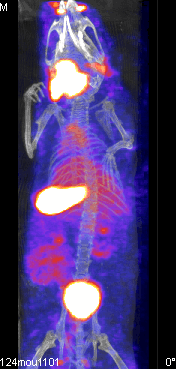

Supplement: Supplementary file 1 — (GIF 2.32 MB) [file 259_2024_6889_MOESM1_ESM.gif]

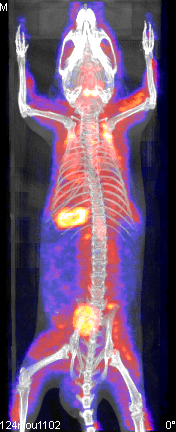

Supplement: Supplementary file 2 — (GIF 3.03 MB) [file 259_2024_6889_MOESM2_ESM.gif]
