# Supplementary material for: Spatial and temporal tracking of multi-layered cells sheet using reporter gene imaging with human sodium iodide symporter: a preclinical study using a rat model of myocardial infarction
Source: Eur J Nucl Med Mol Imaging. 2024 Aug 29;52(1):74–87. doi: 10.1007/s00259-024-06889-2 (PMC11599416; doi:10.1007/s00259-024-06889-2)

***SUPPLEMENTARY MATERIALS FOR:***

**Spatial and temporal tracking of multi-layered cells sheet using reporter gene imaging with human sodium iodide symporter: a preclinical study using a rat model of myocardial infarction**

Kentaro Otani^1^, Tsutomu Zeniya^2^, Hidekazu Kawashima^3^, Tetsuaki Moriguchi^4^, Atsushi Nakano^5^, Chunlei Han^6^, Shunsuke Murata^7^, Kunihiro Nishimura^7^, Kazuhiro Koshino^8^, Kenichi Yamahara^9^, Masayuki Inubushi^10^, Hidehiro Iida^6^

^1^ Department of Molecular Pharmacology, National Cerebral and Cardiovascular Center Research Institute, Osaka, Japan

^2^ Graduate School of Science and Technology, Hirosaki University, Aomori, Japan

^3^ Radioisotope Research Center, Kyoto Pharmaceutical University, Kyoto, Japan

^4^ Tandem Accelerator Complex (UTTAC) University of Tsukuba, Ibaraki, Japan

^5^ Department of Research Promotion and Management, National Cerebral and Cardiovascular Center Research Institute, Osaka, Japan

^6^ Turku PET Centre, University of Turku, Turku, Finland

^7^ Department of Preventive Medicine and Epidemiology, National Cerebral and Cardiovascular Center Research Institute, Osaka, Japan

^8^ Department of Systems and Informatics, Hokkaido Information University, Hokkaido, Japan

^9^ Laboratory of Molecular and Cellular Therapy, Institute for Advanced Medical Sciences, Hyogo Medical University, Hyogo, Japan

^10^ Division of Nuclear Medicine, Department of Radiology, Kawasaki Medical School, Okayama, Japan

**Supplementary Method**

**Isolation and culture of mouse embryonic fibroblasts (MEFs)**

Female WT mice were crossed with male hNIS-Tg mice, and the day after cohabitation was defined as E0.5. Primary hNIS-Tg or WT MEFs were isolated from E13.5 embryos as previously reported [[14](#_ENREF_14)] with minor modifications. In brief, the limbs, tail, visceral tissues, and head of each embryo were dissected and removed, and the remaining tissue was minced and incubated with 0.25% Trypsin-EDTA (#25200, Thermo Fisher Scientific K.K., Tokyo, Japan) for 20 minutes at 37°C. The digested tissues were passed through a Falcon^®^ 70 μm cell strainer (Corning Incorporated, Corning, NY, USA). Cells were plated onto a 60-mm culture dish in 4 ml of DMEM (#11965, Thermo Fisher Scientific K.K.) supplemented with 10% foetal bovine serum, 1×MEM non-essential amino acids (#11140, Thermo Fisher Scientific K.K.), 2 mM L-glutamine (#25030, Thermo Fisher Scientific K.K.), 0.1 mM 2-mercaptoetanol (#137-06862, FUJIFILM Wako Pure Chemical Corporation, Osaka, Japan), 100 U/mL Penicillin-Streptomycin (#15140, Thermo Fisher Scientific K.K.), and 20 mM HEPES (Dojindo Laboratories, Kumamoto, Japan), and maintained at 37°C with 5% CO_2_. For genotyping each embryo, genomic DNA was extracted from the excised head tissue using the DNeasy Blood & Tissue Kit (Qiagen K.K.). Throughout the study, MEFs at passage 3-5 were used.

**Semi-quantitative reverse transcription PCR**

hNIS mRNA expression in MEFs was quantified by semi-quantitative reverse transcription PCR. Total RNA was extracted from WT and hNIS-Tg MEFs using an RNeasy Mini Kit (Qiagen K.K.), and the first-strand complementary DNA was synthesised from 1 μg of total RNA using a QuantiTect® reverse transcription kit (Qiagen K.K.). PCR amplification was performed using a GoTaq® Green Master Mix (Promega K.K., Tokyo, Japan) with the following conditions: 95°C for 2 min, 30 cycles of 95°C for 30 sec, 65°C for 30 sec, 72°C for 20 sec, and 72°C for 5 min. The primers used for semi-quantitative PCR were as follows: *NIS*, forward: 5’-AGCACCTACGAGTACCTGGA-3’, reverse: 5’**-**GCCGGTGCGTAGATTACGAT-3’; *Rplp0*, forward: 5’-GCTTTCTGGAGGGTGTCCG-3’, reverse: 5’-**GTGGGAAGGTGTACTCAGTCTC**-3’.

**Immunocytochemistry of hNIS**

The subcellular distribution of the hNIS protein in hNIS-Tg MEFs was examined by immunocytochemistry, as reported previously [[15](#_ENREF_15)], with minor modifications. In brief, 24 hours before immunocytochemistry, MEFs were seeded at 1×10^5^ cells/dish on a 35-mm-diameter glass-base dish (#3910-035, AGC Techno Glass Co., Ltd., Shizuoka, Japan). The MEFs were rinsed twice with phosphate buffered saline (PBS), fixed in 4% paraformaldehyde (#163-20145, FUJIFILM Wako Pure Chemical) for 10 minutes at room temperature, and permeabilised with 0.1% Triton X-100 in PBS. After blocking with the DAKO protein block (#X0909, Dako, Glostrup, Denmark), MEFs were incubated with a 1:100 dilution of mouse anti-hNIS monoclonal antibody (#MS-1653-P0, Thermo Fisher Scientific K.K.) for 1 hour at room temperature. Then, MEFs were incubated with a 1:200 dilution of Alexa Fluor 488 goat anti-mouse IgG antibody (#A11029, Thermo Fisher Scientific K.K.) for 1 hour at room temperature. After washes with PBS, the nucleuses were stained with DAPI (VECTASHIELD H-1500, Vector laboratories Inc., Burlingame, CA, USA). Fluorescent images were acquired using the BIOREVO BZ-9000 microscope (Keyence Corporation, Osaka, Japan).

**^99m^TcO_4_^–^ uptake assay**

To examine the function of the hNIS protein in hNIS-Tg MEFs, ^99m^TcO_4_^–^ uptake assay was performed as previously described [[16](#_ENREF_16)]. In brief, WT or hNIS-Tg MEFs were seeded at 5×10^4^ cells/well on 24-well culture plates, 24 hours before the assay. On the day of the experiment, ^99m^TcO_4_^–^ was eluted from a ^99^Mo–^99m^Tc generator (Meditec®; Nihon Medi-Physics, Tokyo, Japan), and MEFs were incubated with a culture medium containing 3.7 kBq/500 μL/well of ^99m^TcO_4_^–^ for 5, 30, and 60 minutes at room temperature. After washing twice with ice-cold Hanks’ balanced salt solution, the cells were lysed with 800 μL of 0.1 N NaOH. The radioactivity of the cell lysates was measured using a gamma counter (1480 WIZARD 3, PerkinElmer Inc., Waltham, MA, USA). The percentage of ^99m^TcO_4_^–^ uptake was calculated by dividing the radioactivity of cell lysates by the total amount of radioactivity. The uptake of ^99m^TcO_4_^–^ by MEFs was also examined after pretreatment with 50 μM sodium perchlorate (NaClO_4,_ #198-09252, FUJIFILM Wako Pure Chemical), a competitive inhibitor of hNIS. Assays were performed in quadruplicate using five WT and hNIS-Tg MEFs lines.

**MEFs sheets transplantation**

Two weeks after the surgical procedure, a triple-layered MEFs sheets were transplanted to the infarcted rat heart. The preparation and stacking of MEFs sheets were performed as described previously [[17](#_ENREF_17)]. In brief, WT or hNIS-Tg MEFs were seeded into 35-mm temperature-responsive culture dishes (UpCell, CellSeed Inc., Tokyo, Japan) at 3×10^6^ cells per dish on the day before MEFs sheets transplantation and cultured at 37°C overnight. To detach the MEFs sheet, the culture dish was incubated at room temperature for 40 minutes. A detached MEFs sheet was aspirated with culture media into the tip of a 25-mL pipette and transferred to a new temperature-responsive culture dish. The culture medium was dropped slightly onto the MEFs sheet for spreading. The dish was incubated at 37°C to allow the MEFs sheet to attach to the culture surface. The second MEFs sheet aspirated into the tip of a 25-mL pipette was dropped onto the first MEFs sheet and spread by gently dropping the culture medium. Triple-layered MEFs sheets were prepared by repeating the above procedure. Consequently, 9 hNIS-Tg and 2 WT MEFs sheets were prepared.

**Supplementary Table.** Two-way ANOVA for the ^99m^TcO_4_**^–^** uptake assay

1. WT, ClO_4_^–^ (–) vs. hNIS-Tg, ClO_4_^–^ (­–)

|  | Df | Sum Sq | Mean Sq | F value | p value | Omega Sq | Partial Eta Sq |
| --- | --- | --- | --- | --- | --- | --- | --- |
| Genotype | 1 | 990.401 | 990.401 | 52.083 | <0.001 | 0.512 | 0.685 |
| Time | 2 | 218.709 | 109.354 | 5.751 | 0.009 | 0.095 | 0.324 |
| Interaction | 2 | 214.293 | 107.147 | 5.635 | 0.01 | 0.093 | 0.32 |

1. WT, ClO_4_^–^ (–) vs. WT, ClO_4_^–^ (+)

|  | Df | Sum Sq | Mean Sq | F value | p value | Omega Sq | Partial Eta Sq |
| --- | --- | --- | --- | --- | --- | --- | --- |
| ClO_4_^–^ | 1 | 0.002 | 0.002 | 24.061 | <0.001 | 0.113 | 0.501 |
| Time | 2 | 0.014 | 0.007 | 71.98 | <0.001 | 0.696 | 0.857 |
| Interaction | 2 | 0.001 | 0.001 | 5.435 | 0.011 | 0.044 | 0.312 |

1. hNIS-Tg, ClO_4_^­–^ (–) vs. hNIS-Tg, ClO_4_^­–^ (+)

|  | Df | Sum Sq | Mean Sq | F value | p value | Omega Sq | Partial Eta Sq |
| --- | --- | --- | --- | --- | --- | --- | --- |
| ClO_4_^–^ | 1 | 983.856 | 983.856 | 51.738 | <0.001 | 0.51 | 0.683 |
| Time | 2 | 219.048 | 109.524 | 5.76 | 0.009 | 0.096 | 0.324 |
| Interaction | 2 | 213.958 | 106.979 | 5.626 | 0.01 | 0.093 | 0.319 |

1. WT, ClO_4_^–^ (+) vs. hNIS-Tg, ClO_4_^­–^ (+)

|  | Df | Sum Sq | Mean Sq | F value | p value | Omega Sq | Partial Eta Sq |
| --- | --- | --- | --- | --- | --- | --- | --- |
| Genotype | 1 | 0.023 | 0.023 | 47.787 | <0.001 | 0.42 | 0.666 |
| Time | 2 | 0.017 | 0.008 | 17.369 | <0.001 | 0.294 | 0.591 |
| Interaction | 2 | 0.002 | 0.001 | 1.958 | 0.163 | 0.017 | 0.14 |

**Supplementary Fig.S1**

**
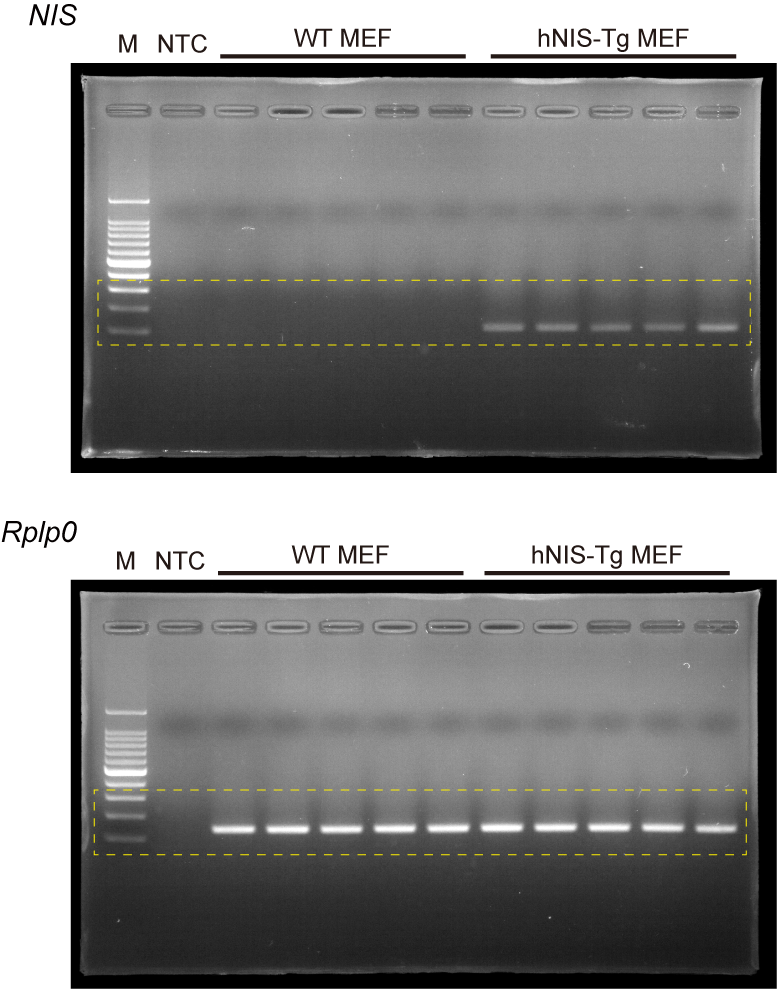
**

Uncropped gel images from semiquantitative PCR. The yellow dotted line indicates the sections used in **Fig. 2A**. NTC, No-template control

**Supplementary Fig.S2**

**
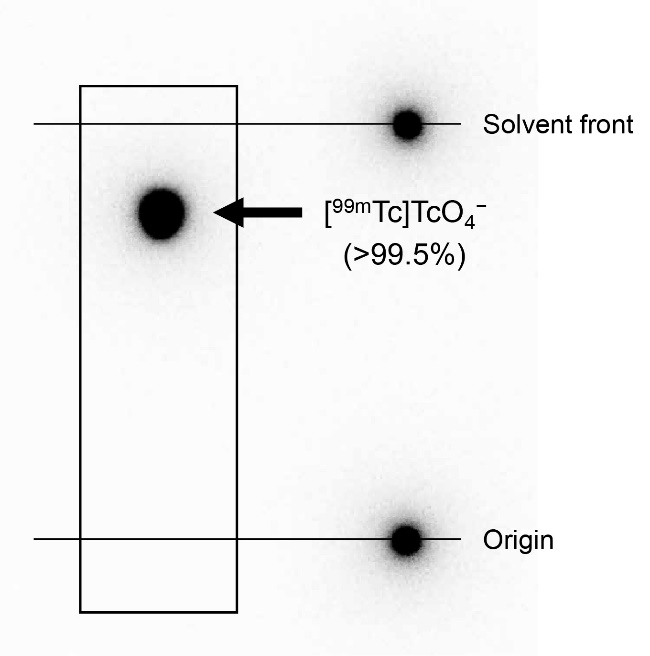
**

The result of radio-TLC analysis. Under this condition, the hydrolyzed-reduced ^99m^Tc (^99m^Tc-colloid) retains at original position (Rf = 0).

**Supplementary Fig.S3**

**
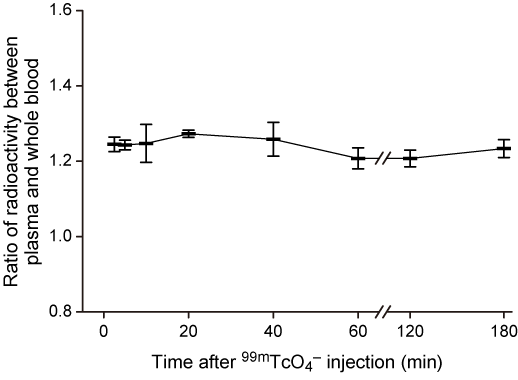
**

The ratio of radioactivity between plasma and whole blood throughout the experiments

**Supplementary Fig.S4**

**
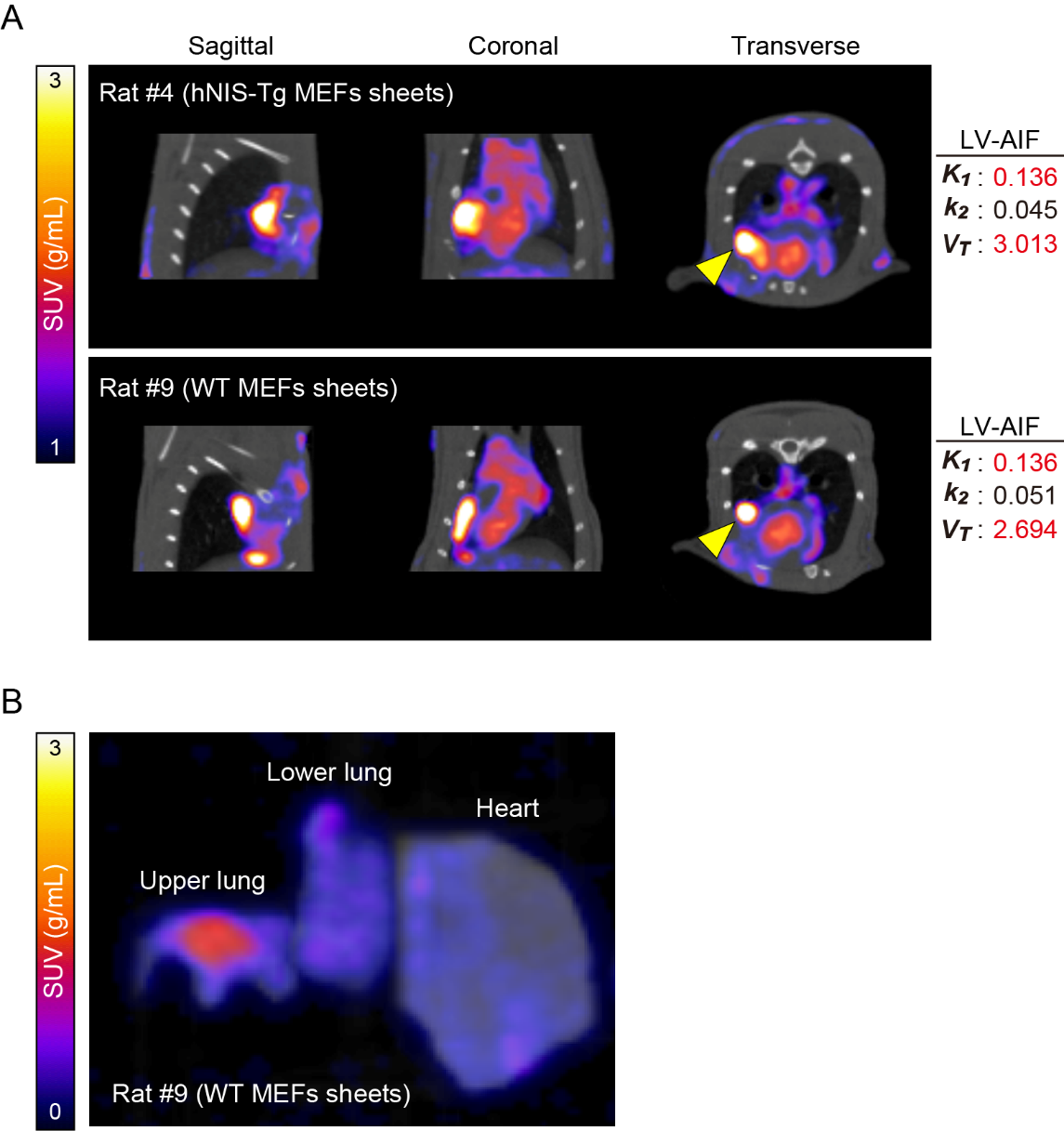
**

Nonspecific accumulation of ^99m^TcO_4_^-^ in the lung. (**A**) Sagittal, coronal, and transverse SPECT/CT images of **Rat #4** and **Rat #9** 40–60 minutes after ^99m^TcO_4_^-^ injection. The yellow arrowheads in the figure indicate the site of non-specific ^99m^TcO_4_^-^ accumulation. (**B**) SPECT image of the excised heart and lung of **Rat #9**.

**Supplementary Movie S1.** Whole-body maximum intensity projection (MIP) images of the WT mouse.


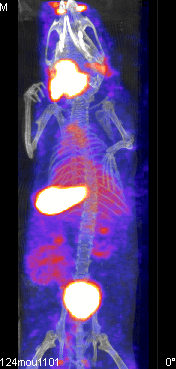


**Supplementary Movie S2.** Whole-body maximum intensity projection (MIP) images of the hNIS-Tg mouse.


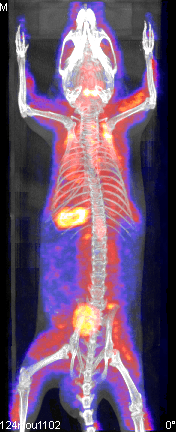

Supplement: Supplementary file 3 — (DOCX 10.5 MB) [file 259_2024_6889_MOESM3_ESM.docx]
